# B cell sub-types following acute malaria and associations with clinical immunity

**DOI:** 10.1186/s12936-016-1190-0

**Published:** 2016-03-03

**Authors:** Richard T. Sullivan, Isaac Ssewanyana, Samuel Wamala, Felistas Nankya, Prasanna Jagannathan, Jordan W. Tappero, Harriet Mayanja-Kizza, Mary K. Muhindo, Emmanuel Arinaitwe, Moses Kamya, Grant Dorsey, Margaret E. Feeney, Eleanor M. Riley, Chris J. Drakeley, Bryan Greenhouse, Richard Sullivan

**Affiliations:** Department of Medicine, University of California San Francisco, Box 0811, San Francisco, CA 94110 USA; Infectious Disease Research Collaboration, Tororo, Uganda; Department of Immunology and Infection, London School of Hygiene and Tropical Medicine, London, UK; Centers for Disease Control and Prevention, Atlanta, GA USA; Makerere University Medical School, Kampala, Uganda

**Keywords:** Atypical memory B cells, Transitional B cells, Plasmablasts, Plasma cells *Plasmodium falciparum*, Malaria, Immunity

## Abstract

**Background:**

Repeated exposure to *Plasmodium falciparum* is associated with perturbations in B cell sub-set homeostasis, including expansion atypical memory B cells. However, B cell perturbations immediately following acute malaria infection have been poorly characterized, especially with regard to their relationship with immunity to malaria.

**Methods:**

To better understand the kinetics of B cell sub-sets following malaria, the proportions of six B cell sub-sets were assessed at five time points following acute malaria in four to 5 years old children living in a high transmission region of Uganda. B cell sub-set kinetics were compared with measures of clinical immunity to malaria—lower parasite density at the time of malaria diagnosis and recent asymptomatic parasitaemia.

**Results:**

Atypical memory B cell and transitional B cell proportions increased following malaria. In contrast, plasmablast proportions were highest at the time of malaria diagnosis and rapidly declined following treatment. Increased proportions of atypical memory B cells were associated with greater immunity to malaria, whereas increased proportions of transitional B cells were associated with evidence of less immunity to malaria.

**Conclusions:**

These findings highlight the dynamic changes in multiple B cell sub-sets following acute, uncomplicated malaria, and how these sub-sets are associated with developing immunity to malaria.

## Background

Malaria caused by *Plasmodium falciparum* continues to cause over a half million deaths each year, with children being disproportionately affected [1]. Children suffer the greatest morbidity and mortality from malaria since immunity to malaria takes years to develop, increasing with age and exposure [2, 3]. One manifestation of acquired immunity to malaria is control of blood stage parasites, resulting in lower parasite densities and lack of febrile symptoms of disease [4–6]. Antibodies have been shown to be an important mediator of this blood stage immunity [7–10].

Effective B cell and antibody responses to *Plasmodium* infection generally develop only after years of repeated exposure, likely due to immune immaturity of the host and antigenic variation of parasites [8–12]. Another hypothesis for the slow development of immunity is that *Plasmodium* infection may interfere with B cell development and maintenance of memory responses [13–17]. After initial maturation in the bone marrow, B cells pass through a series of developmental differentiation stages, many of which can be detected in the peripheral blood. Transitional B cells emerge from the bone marrow and mature into naïve B cells prior to antigen exposure. After antigen exposure, B cells in secondary lymphoid organs differentiate into class-switched classical memory B cells (MBCs) non-class switched ‘innate-like’ MBCs and antibody-secreting plasmablasts/plasma cells [18]; these cells can be detected in blood as they migrate to other secondary lymphoid organs and tissues. Exposure to *Plasmodium* alters the distribution of these B cell sub-sets, and has been associated with an expansion of ‘atypical’ MBCs in individuals living in malaria-endemic areas [13–15, 19]. Atypical MBCs are class-switched but lack the classical MBC marker CD27, and unlike classical MBCs, do not appear to readily produce antibodies [13, 20, 21]. This functional difference has led to the hypothesis that atypical MBCs may be ‘exhausted’ and may interfere with development of effective immunity [13, 21]. On the other hand, higher circulating proportions of atypical MBCs and immunity to malaria are both associated with increasing age and *P. falciparum* exposure [13, 14, 22–24]. Thus, the relationship between atypical MBCs and immunity to malaria remains unclear.

B cell sub-sets generated during malaria episodes may indicate which B cells are associated with developing immunity. Various studies have described multiple B cell sub-sets in people exposed to varying levels of malaria [11, 13, 14, 20–23, 25, 26], but the kinetics of B cell responses following malaria have not been well described in humans. One study tracked the kinetics transitional B cells following malaria and found that the relative proportion of these cells increased following malaria [19]. Studies of experimental infection of mice with *Plasmodium chabaudi* have found that newly differentiated plasmablasts only circulate in the blood for a short time following primary or secondary infection while other sub-sets such as transitional, naïve B cells and MBCs fluctuate greatly but remain readily detectable in the peripheral blood [26]. These findings suggest that there are likely to be dynamic changes in the composition of the B cell pool both during and following acute malaria in humans, and that these changes may be reflected in the peripheral blood. Here, the kinetics of six distinct sub-sets of B cells were evaluated during and after treatment for symptomatic malaria, and sub-set proportions were evaluated for associations with measures of immunity to malaria.

## Methods

### Study cohort

Samples were obtained from participants between 4.6 and 5.0 years of age enrolled in the Tororo Child Cohort (TCC) study in Tororo, Uganda, an area of intense malaria transmission (annual entomological inoculation rate in the region estimated at 125 infectious bites per person year) [27]. Cohort details have been described elsewhere [28–30], but in brief, TCC children were enrolled at infancy (mean 2.7 months old) and followed to 5 years of age at a dedicated study clinic, providing all medical care. Febrile participants (>38 °C tympanic temperature or reported fever in the previous 24 h) were tested for *P. falciparum* parasites by thick blood smear. Febrile participants that had any detectable parasites by thick blood smear were diagnosed with symptomatic malaria and treated with an artemisinin-based combination therapy. Participants also had monthly blood smears to assess parasitaemia regardless of symptoms. For this study, 38 consecutive children were enrolled that presented with symptomatic malaria, with parasite densities greater than 2000 parasites/µl. Asymptomatic parasitaemia was defined by parasitaemia in afebrile participants occurring at least 14 days after and five days before a malaria episode.

### Ethical permission

All study participants or their parents or guardians provided individual written informed consent. Approval was granted by the Uganda National Council of Science and Technology, the Makerere University Research and Ethics Committee, the University of California, San Francisco Committee on Human Research,

### Whole blood staining

**Fig. 1:**
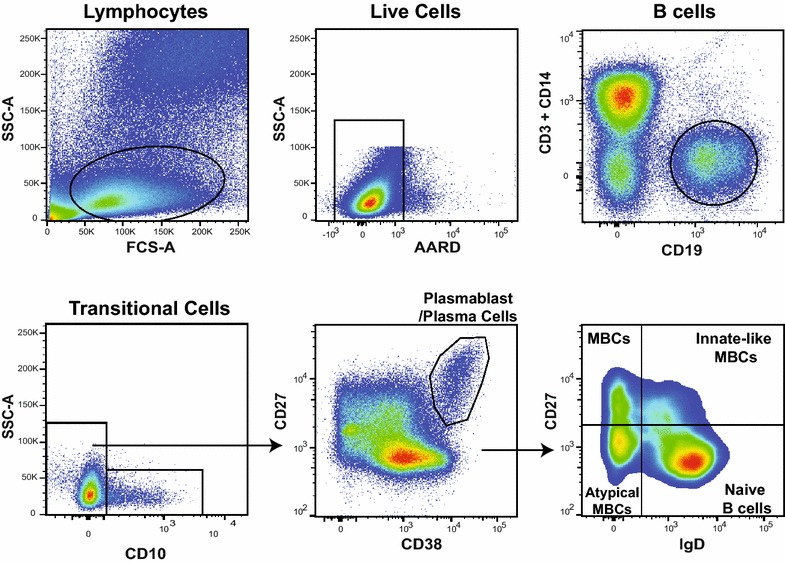
Representative flow cytometry dot plot and gating hierarchy used to define B cell sub-types using cell markers CD3/14, CD19, CD10, CD38, CD27, and IgD. Using these markers, six CD19+B cell sub-sets are defined as: transitional (CD10^+^ CD27^−^ IgD^+^), naïve (CD10^−^ CD27^−^ IgD^+^), innate-like MBCs (CD10^−^ CD27^+^ IgD^+^), classical MBCs (CD10^−^ CD27^+^ IgD^−^), atypical MBCs (CD10^−^ CD27^−^ IgD^−^), and plasmablasts/plasma cells (CD10^−^ CD27^hi^ CD38^hi^ IgD^−^)

A half ml of whole blood was collected through finger prick or phlebotomy. Whole blood was washed with PBS and then labelled for 30 min with antibodies against CD3 (clone UCHT1), CD14 (clone M5E2), CD19 (clone HIB19), CD10 (clone HI10a), CD38 (clone HIT2), CD27 (clone O323), IgG (clone G18-145) (all BioLegend), and IgD (clone IA6-2) (BD Biosciences) (see Fig. 1 for gating strategy). Due to limitations in the number of stable fluorophores available in this assay, CD21 could not be included for analysis. However, previous studies have demonstrated that the majority of IgD- CD27-B cells are also CD21- and have similar expression profiles [20, 21, 31–36]. Whole blood was treated with Lyse/Fix buffer (BD Biosciences), to remove red blood cells and fix stained cells. Cells were then stored in PBS at −80 °C until stained and processed on an LSRII (Fig. 1). Prior to the initiation of this study, the stability of the fluorescent markers was verified with B cell sub-sets readily distinguishable up to six weeks after staining when stored at −80 °C. All samples were run on the LSRII within four weeks of staining. As inclusion criteria for this analysis, a minimum of 10,000 B cell events were collected, with each sub-set consisting of at least 200 events, from each time point. In order to limit variations or batch effects between LSRII cytometer uses, all flow cytometric data were collected using rainbow beads to equilibrate relative fluorescence.

### Statistical analysis

Changes in B cell sub-type proportions between day 0 and other days were tested using the Wilcoxon signed rank test. Linear trends in proportion during follow-up were evaluated using generalized estimating equations [37] to account for repeated measures in the same individual. To test the hypothesis that clinical immunity was associated with proportions of B cell sub-sets, the proportions during and following malaria with parasite density on day 0, with lower parasite density indicating greater immunity, and with the number of asymptomatic parasitaemia episodes in the prior 180 days, with more episodes indicating greater immunity. Parasite density was evaluated as a binary variable based on the median parasite density of 23,080 parasite/μl. Asymptomatic parasitaemia was evaluated as an ordinal variable of 0, 1 or greater than 1 episode in the last 180 days, with a linear trend across the three categories evaluated. Associations between the clinical metrics and B cell sub-types were evaluated using generalized estimating equations. To account for the potential confounding effects of prior exposure, a multivariate analysis including the incidence of malaria between 12 and 24 months of age was performed, when the effect of acquired immunity was likely to be minimal as an estimate of prior exposure.

## Results

### Participant characteristics

For this study, 38 children between the ages of 4.6 and 5.0 years were consecutively enrolled at the time of presentation with symptomatic malaria and followed them for 28 days (Table 1). The age range was intentionally kept narrow since age may strongly affect immune responses and clinical immunity. All children lived in Tororo, a district of Uganda with intense perennial malaria transmission, and were enrolled from a larger cohort study in which they had been followed since at least 10 months old [27, 29]. Table 1 shows the range of parasite densities at baseline, the number of episodes of symptomatic malaria and asymptomatic parasitaemia in the preceding 180 days, and the proportion of participants who went on to have another bout of clinical malaria during the 28-day active surveillance period following treatment.

**Table 1:** Characteristics of study participants

Participants’ characteristic (n = 38)	Mean	Range
Age (years)	4.8	4.6–5.0
Parasite density on day 0 (parasites/µl)	23,080^a^	2000–180,000
Asymptomatic parasitaemia episodes in the prior 180 days	0.5	0–5
Symptomatic malaria episodes in the prior 180 days	3.8	1–8
Proportion of participants with recurrent malaria^b^	10/38	

^a^Median

^b^Recurrent malaria as defined symptomatic malaria in the 28 days following enrolment

### Atypical MBCs and transitional B cell proportions increased, whereas plasma cell and naïve B cell proportions decreased, following malaria

**Fig. 2:**
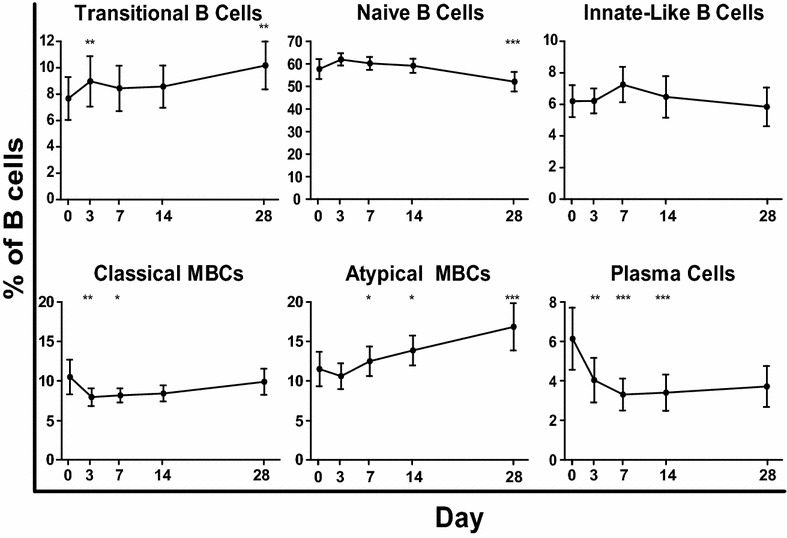
B cell kinetics following acute malaria. *Graphs* show the mean proportion, with bars denoting 95 % confidence intervals, of each B cell sub-type. *Asterisk*, *double asterisk* and *triple asterisk* indicate a significant difference in proportion from day 0 as assessed by the Wilcoxon rank sum test (p < 0.05, 0.01 and 0.001, respectively). Day 0 n = 36, day 3 n = 36, day 7 n = 38, day 14 n = 36, and day 28 n = 36

**Fig. 3:**
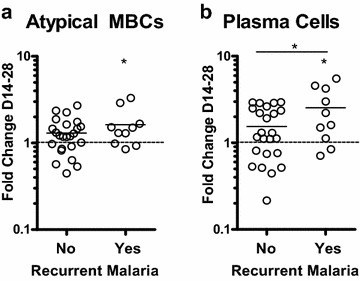
Fold change in atypical MBC and PC proportions from day 14 to day 28 in participants who did and did not have a recurrent malaria episode between days 14 and 28 of follow-up. **a** Fold changes in atypical MBC proportions. Participants with a second malaria episode had a significant increase in atypical MBC proportions from day 14 to when they presented with a recurrent malaria episode when tested by Wilcoxin signed-rank test, but this increase was not significantly higher than the fold changes in participants who did not have a recurrent malaria episode. **b** Fold changes in PC proportions. Participants with a recurrent malaria episode had a significant increase in PC proportions from day 14 to when they presented with the recurrent malaria episode by Wilcoxin signed-rank test. Additionally, this increase was significantly higher than the changes in participants who did not have a recurrent malaria episode, as assessed by Wilcoxin rank sum test and indicated by *asterisk* above *solid bar*

In order to characterize the kinetics of B cell sub-sets following acute malaria, 6 B cell sub-sets were characterized from whole peripheral blood collected at the time of malaria diagnosis (day 0), and on days 3, 7, 14, and 28 following treatment. Flow cytometry was used to measure the relative proportions of: transitional B cells (CD19+CD10+), naïve B cells (CD19+CD10−IgD+CD27−), innate-like MBCs (CD19+CD10−IgD+CD27+), classical MBCs (CD19+CD10−IgD−CD27+), atypical MBCs (CD19+CD10−IgD−CD27−), and plasmablasts/plasma cells (CD19+CD10−IgD−CD27^hi^CD38^hi^) (Fig. 1). The proportions of atypical MBCs and transitional B cells both increased in the 28 days following malaria (Fig. 2). Atypical MBC proportions increased from a mean of 11.8 to 17.3 %, day 0 to day 28 (p < 0.001 for trend). In order to distinguish whether this trend represented an increase in atypical MBC proportions following malaria versus a transient decrease at the time of malaria, the proportions of atypical MBCs in participants that had a second malaria episode during the 28-day study observation period were evaluated. In participants that had a second malaria episode during the 28-day study observation period, the proportions of atypical MBCs increased further following the second malaria episode, from 11.5 to 16.0 % (p = 0.04) (Fig. 3a), providing evidence against a transient decrease at the time of malaria. Transitional B cell proportions also increased following malaria, from a mean of 7.7 % on day 0 to 10.0 % on day 28 (p < 0.001 for trend), however no additional increase in transitional B cells was observed in participants that had a second malaria episode during the 28-day study observation period.

In contrast, proportions of plasmablasts/plasma (PC) cells and naïve B cells decreased following malaria. Mean PC proportion was highest at 6.1 % on day 0 and then rapidly declined to 3.3 % by day 7 (p = 0.002). Participants with a second malaria episode during the 28-day study had increased PC proportions following second episode diagnosis (Fig. 3b), and this increase in PC proportion was significantly higher in participants with a second malaria episode compared to the rest of the cohort. This suggests that plasmablasts, known to only circulate briefly in the peripheral blood, can be readily detected in significant numbers in the peripheral blood at the time of symptomatic malaria. Naïve B cell proportion decreases were moderate, from 57 to 51 % from day 0 to day 28 (p = 0.016). Classical memory B cell proportions had a transient drop on days 3 and 7 only, but otherwise remained unchanged throughout observation. No significant fluctuations in innate-like MBC proportions was detected over the 28-day study.

### Atypical memory B cell proportions were higher among children with evidence of immunity to malaria

**Fig. 4:**
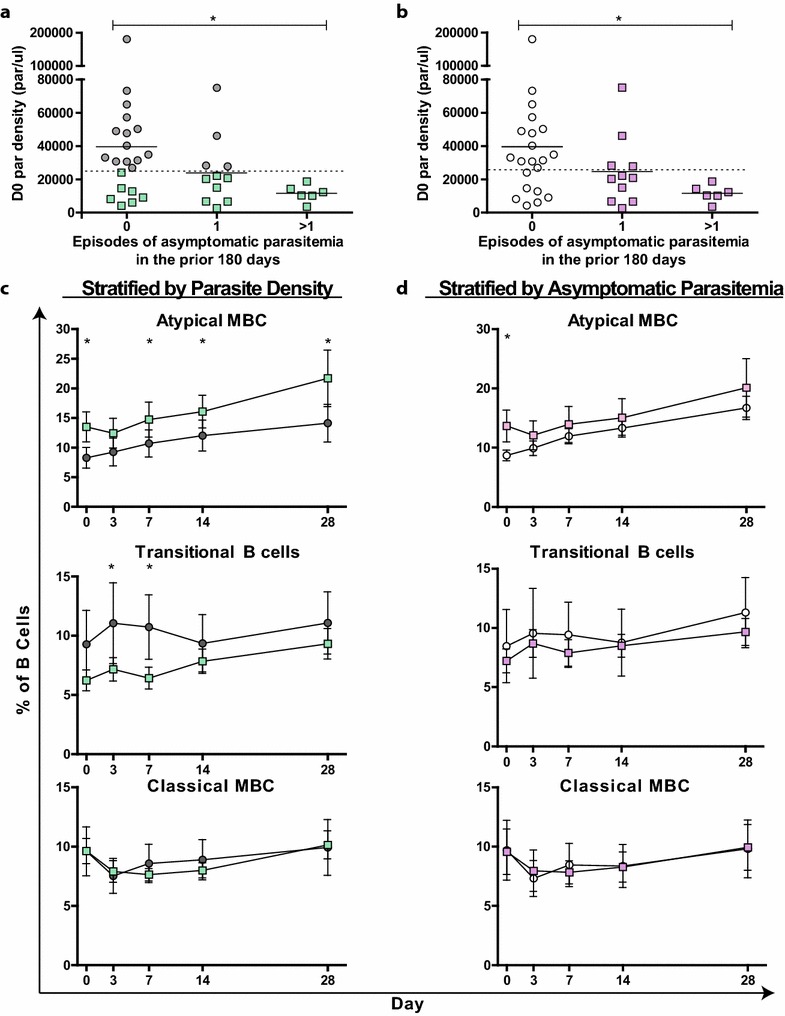
Kinetics of atypical MBC, transitional and classical MBC proportions in participants categorized by parasite density and recent asymptomatic parasitaemia. **a** and **b**: participants were separated by day 0 parasite densities of greater than or less than 23,080 parasite/µl (*green boxes*, **a**), or by having at least one recently documented case of asymptomatic parasitaemia (*purple boxes*, **b**). **c** *Lines* were based on categories of parasite density from (**a)**, and depict proportions of B cell sub-sets over the 28-day observation period following malaria (day 0). **d** *Lines* were based on category of asymptomatic parasitaemia from (**b)**, and depict proportions of B cell sub-sets over the 28-day observation period following malaria (day 0). **c** and **d** *Lines* represent mean proportion for each group, with *bars* denoting 95 % confidence intervals. *Asterisk*, *double asterisk* and *triple asterisk* indicate a significant difference between groups at that timepoint as assessed by Wilcoxon rank sum test (p < 0.05, 0.01 and 0.001, respectively, ns = no significant difference)

Higher proportions of atypical MBCs and immunity to malaria are both positively associated with age and *P. falciparum* exposure. Therefore, this study sought to test if proportions of atypical MBCs were associated with measures of developing immunity to malaria. None of the participants included in this study were fully immune to symptomatic malaria, since malaria was the primary inclusion criteria for enrollment, however a range of clinical immunity was noted in these children sharing a similar age range. Acquired immunity to malaria manifests as better control of blood stage parasites, resulting in lower parasite density and a decreased probability of infection developing into symptomatic malaria [2, 3, 38]. Therefore, participants were grouped based on parasite density at the time of malaria (Fig. 4a) and documented asymptomatic parasitaemia in the prior 180 days (Fig. 4b). A history of asymptomatic parasitaemia was associated with lower parasite density at presentation with malaria, as would be expected if both were indications of clinical immunity.

When participants were grouped based on median parasite densities (23,080 parasites/µl) at the time of malaria, participants with lower parasite density had higher proportions of atypical MBCs during the 28 days following malaria (Table 2). A similar association was observed between the geometric mean parasite density over the previous 180 days and the proportion of atypical MBC over the 28 days following malaria (p = 0.02), indicating some temporal stability in the clinical phenotype of control of blood-stage parasites. Participants with more documented episodes of asymptomatic parasitaemia within the prior 180 days were also found to have higher proportions of atypical MBCs following malaria. These associations remained significant and largely unchanged after adjusting the analysis for varied *P. falciparum* exposure. Further, these associations were consistent throughout the 28 days following malaria (Fig. 4c, d). Proportions were more strongly associated with day 0 parasite density than with asymptomatic parasitaemia, and in a multivariate analysis including both of these correlated measures, only parasite density remained significant (p < 0.001). Together, these findings indicate that measures of greater immunity to malaria were associated with increased proportions of atypical MBCs in the peripheral blood following malaria.

**Table 2:** Associations between measures of clinical immunity and proportions of B cell sub-sets

B cell sub-set	Day 0 parasite density <median^a^ Mean difference in % proportion (p value)		Asymptomatic parasitaemia prior 180 days^b^ Mean difference in % proportion (p value)	
	Unadjusted	Adjusted^c^	Unadjusted	Adjusted^c^
Atypical MBCs	*4.5 (0.001)*	*5.1 (<0.001)*	*1.9 (0.05)*	*3.8 (0.04)*
Transitional	*−2.6 (0.08)*	*−3.6 (0.02)*	−0.6 (0.5)	−1.1 (0.3)
Classical MBCs	−0.2 (0.8)	0.4 (0.7)	−0.6 (0.3)	−0.2 (0.7)

Associations were measured using all sampling time points from each participant compared to either day 0 parasite densities or prior episodes of asymptomatic parasitaemia in previous 180 days. Day 0 parasite density ≤ 23,080 parasites/μl n = 20 and > 23,080 parasites/μl n = 18. Of the 38 participants in this study, ten had one or more episodes of asymptomatic parasitaemia in the previous 180 days

Results in italics have a significant analysis adjusted p value

^a^Median parasite density = 23,080 parasites/μl

^b^Linear relationship between 0, one and more than one episode

^c^Analysis adjusted for *P. falciparum* exposure, as estimated by the incidence of malaria in that child between 12 and 24 months of age

### Transitional B cell proportions were lower among children with evidence of immunity to malaria

In contrast to atypical MBCs, transitional B cell proportions were lower in participants that had parasite density less than the median at the time of malaria (Table 2), but no significant association with asymptomatic parasitaemia was found. The association with parasite density remained significant after adjusting the analysis for varied *P. falciparum* exposure. Further, this association was consistent throughout the 28 days following malaria (Fig. 4c). A similar association was found between the geometric mean parasite density over the previous 180 days and the proportion of transitional B cells following malaria. This finding indicates that participants with evidence of greater immunity to malaria tend to have lower proportions of transitional B cells in the peripheral blood following malaria.

### Associations between other B cell sub-types and immunity to malaria

Proportions of circulating plasma cells/plasmablasts rapidly decreased following malaria (day 0), but were not significantly associated with measures of immunity to malaria. There were no significant associations between measures of immunity to malaria and the proportions of classical MBCs, innate-like MBCs or naïve B cells.

## Discussion

This study investigated the kinetics of different B cell sub-sets at the time of and following symptomatic malaria and then evaluated whether proportions of these sub-sets were associated with measures of immunity to malaria. The proportions of atypical MBCs and transitional B cells both increased during the 28 days following symptomatic malaria, whereas naïve B cell proportions declined slowly and circulating PCs proportions declined rapidly following treatment of malaria, increasing again in participants who had a second episode of malaria. Notably, children with evidence of greater immunity to malaria had higher proportions of atypical MBCs and lower proportions of transitional B cells following malaria, and these associations remained stable over the 28 days following malaria. These findings suggest that children with higher proportions of these cells during and following malaria have improved immune responses to *P. falciparum* infection.

People living in malaria-endemic regions have increased proportions of atypical MBCs, with these proportions increasing with age and with cumulative *Plasmodium* exposure [11, 13, 14]. Atypical MBCs have been characterized as having upregulated inhibitory pathways compared to classical MBCs [13, 20, 21]. Despite one report suggesting serum antibodies may be produced by atypical MBCs [22], other studies have reported that atypical MBCs have a poor capacity for differentiation and antibody production [13, 20, 21]. With no direct evidence for an alternative function for atypical MBCs, these cells have been hypothesized to represent a dysfunctional or ‘exhausted’ phenotype. However, atypical MBC proportion, like immunity to symptomatic malaria, is associated with age and transmission intensity [20, 24]. This study was not able to evaluate a causal relationship between atypical MBCs and immunity to malaria, but it demonstrates that the accumulation of atypical MBCs in the peripheral blood during and after malaria is associated with measures of clinical immunity. This association did not appear to merely be an artifact of age, since all participants in this study were nearly the same age, or of varied *P. falciparum* exposure, as results were unchanged in a multivariate analysis including estimates of exposure. One possibility is that the ability to sustain asymptomatic infections, which tend to be of longer duration than treated symptomatic malaria infections, leads to higher proportions of atypical MBCs due to chronic antigen exposure. Alternatively, it may be that higher parasite densities seen in less immune individuals drive down the proportion of atypical MBCs, possibly as a result of apoptosis or homing of atypical MBCs to tissues. It is also possible that atypical MBCs contribute, in an as-yet undefined manner, to anti-malarial immunity. Further studies are necessary to determine if atypical MBCs are causally associated with immunity to malaria.

Increases in transitional B cell proportions following malaria have been previously noted, and this expansion was hypothesized to be due to disruption of B cell homeostasis [19]. This study replicates the finding of increased proportions of circulating transitional B cell following malaria. In addition, this study found participants with greater parasite density at the time of malaria had higher proportions of transitional B cells. It is possible that this association could be driven by greater *P. falciparum* induced, non-specific, polyclonal B cell activation [39] and apoptosis [40] in individuals with higher parasite burdens, leading to a homeostatic expansion of transitional B cells. It is also possible that transitional B cells function in an immunoregulatory capacity [41], similar to IL-10 producing T cells, in the context of malaria [42]. Transitional B cells from healthy US adults have been shown to secrete IL-10, a potent immunoregulatory cytokine, following CpG stimulation, and regulate T cell responses in vitro through IL-10 secretion [41, 43, 44]. Further studies would be necessary in order to determine whether higher proportions of transitional B cells causally interfere with immunity to malaria, are a result of inadequate immune control of parasites, or whether expansion of this sub-set is associated with increased parasite densities for other reasons.

Outside of vaccine trials, plasmablasts/plasma cells can be difficult to characterize due to their paucity in the peripheral blood and highly synchronous migration to the bone marrow. Development and migration of PCs has been characterized in experimental infection of mice with *P. chabaudi* [9, 45, 46], but is not well described in humans. Although difficult to assess, these cells provide a snapshot of effector B cell responses formed in response to recent exposure. This study demonstrates that the day a person presents with symptomatic malaria may be an optimum time to characterize plasmablasts/plasma cells in peripheral blood. Given the findings, it may be possible in future studies to assess responding plasmablasts formed in response to acute infection and provide insight into emerging B cell responses to natural infection.

Some unique strengths of this study are the detailed longitudinal follow up of participants, allowing characterization of relevant clinical outcomes and the measurement of numerous B cell sub-type proportions at multiple timepoints during and following an episode of symptomatic malaria. By evaluating proportions of these sub-types at the time of malaria and following treatment, the potential confounding effects of the duration of time since malaria, which can affect the proportions of different cell types, was limited. Similarly, by evaluating children in a very narrow age range, the potential confounding effect of age, which is associated with immunity to malaria and proportions of B cell sub-types, was reduced [11, 13]. The extensive longitudinal data available on study participants allowed the evaluation and adjustment for the individual’s varied exposure to *P. falciparum*, an important consideration when evaluating immunity.

A limitation of this study is the inability to determine whether associations between clinical phenotypes and proportions of B cell sub-sets represent a direct effect of these cell types. In addition, evaluation was limited to B cell sub-sets trafficking through peripheral blood which, while clearly an important compartment for interaction with a blood stage pathogen, may not represent proportions in other important compartments such as secondary lymphoid organs or peripheral tissues. There is evidence using experimental mouse infection with *P. chabaudi* that memory B cell responses circulating in the blood reflected the general composition of B cells in peripheral tissues [26]. Since peripheral lymphoid tissues are not easily sampled, understanding circulating responses may provide the closest accessible insights to general B cell responses and homeostasis in humans. Another caveat to this study is that increased or decreased B cell sub-set proportions could be a result of perturbations in another B cell sub-set, e.g., observed decreases in circulating naïve B cell proportions could be the result of increases in transitional cell proportions. Consistent with methods from several earlier prior studies [11, 13, 14, 22, 47], the design of this study only allowed for measurement of relative proportions and not absolute B cell counts; future studies may want to account for changes in absolute numbers of cells as well as proportions. Additional studies focusing on the mechanisms of action of atypical MBCs and transitional B cells are also needed to provide insight into the causal relationships underlying the associations observed.

## Conclusions

The findings of this study highlight the importance of investigating the dynamics of multiple B cell sub-sets, including consideration of the timing of these measurements with respect to malaria, in order to understand the contribution of the humoral immune system in developing immunity to malaria. Infection by *P. falciparum* greatly affects B cell homeostasis, but further research is necessary to understand the full implications of these changes. Since most vaccines are designed to induce a protective B cell response that can be sustained over time, it is vital to understand consequences of *P. falciparum* infection on the humoral immune system, memory formation and maintenance of memory.
