# Erratum to: B cell sub-types following acute malaria and associations with clinical immunity

**DOI:** 10.1186/s12936-016-1236-3

**Published:** 2016-03-31

**Authors:** Richard T. Sullivan, Isaac Ssewanyana, Samuel Wamala, Felistas Nankya, Prasanna Jagannathan, Jordan W. Tappero, Harriet Mayanja-Kizza, Mary K. Muhindo, Emmanuel Arinaitwe, Moses Kamya, Grant Dorsey, Margaret E. Feeney, Eleanor M. Riley, Chris J. Drakeley, Bryan Greenhouse

**Affiliations:** Department of Medicine, University of California San Francisco, Box 0811, San Francisco, CA 94110 USA; Infectious Disease Research Collaboration, Tororo, Uganda; Department of Immunology and Infection, London School of Hygiene and Tropical Medicine, London, UK; Centers for Disease Control and Prevention, Atlanta, GA USA; Makerere University Medical School, Kampala, Uganda

## Erratum to: Malar J (2016) 15:139 DOI 10.1186/s12936-016-1190-0

During production of the original article [1], an error occurred which resulted in the duplication of Richard T. Sullivan’s name within the author list. The original article [1] has been updated to present the correct author list, which also appears in this erratum.
